# MRI-Based Deep Learning Guides Multi-Omics Discovery of NBPF4 as a Therapeutic Target for Breast Cancer Lymph Node Metastasis

**DOI:** 10.34133/research.1402

**Published:** 2026-08-24

**Authors:** Dianqi Cai, Haoxuan Huang, Zijun Chen, Chao Zhu, Zehui Li, Ming Chen, Zhiyi Guo, Jie Liu, Gengxi Cai, Wenjun Mao, Jianguo Lai

**Affiliations:** ^1^ Guangdong Provincial People’s Hospital, Guangdong Academy of Medical Sciences, Southern Medical University, Guangzhou, China.; ^2^ Guangdong Provincial People’s Hospital Ganzhou Hospital, Ganzhou Municipal Hospital, Ganzhou Innovation Center, National Regional Medical Center, Gannan Medical University, Ganzhou, China.; ^3^ The Sixth Affiliated Hospital, Sun Yat-sen University, Guangzhou, Guangdong Province, China.; ^4^ Department of Urology, The Third Affiliated Hospital (the First Hosptial of Nanchang), Jiangxi Medical College, Nanchang University, Nanchang 330006, Jiangxi, China.; ^5^ Department of Clinical Laboratory, The First Affiliated Hospital of Nanchang University, Nanchang, China.; ^6^ Department of Breast Cancer, Foshan Women and Children Hospital Affiliated to Guangdong Medical University, Foshan, China.; ^7^ The First People’s Hospital of Foshan (Foshan Hospital Affiliated to Southern University of Science and Technology), School of Medicine, Southern University of Science and Technology, Guangdong, China.; ^8^ Department of Thoracic Surgery, The Affiliated Wuxi People’s Hospital of Nanjing Medical University, Wuxi People’s Hospital, Wuxi Medical Center, Nanjing Medical University, Wuxi, China.; ^9^ Wuxi College of Clinical Medicine, Nanjing Medical University, Wuxi, China.

## Abstract

Deep learning models are increasingly used to analyze medical images, but their “black box” nature makes it hard to understand the underlying biology and slows down the development of targeted treatments. To tackle this, we built a multi-step approach that combines deep learning analysis of breast magnetic resonance imaging (MRI) with several types of molecular data, including gene activity, protein levels, and genetic information, along with laboratory experiments. Our MRI-based deep learning model accurately predicted whether breast cancer had spread to lymph nodes, and it performed consistently across 3 separate groups of patients. Causal inference using double least absolute shrinkage and selection operator (LASSO) and causal forest double machine learning established a significant effect of *NBPF4* expression on the imaging-defined high-risk phenotype, independent of genomic confounders. When we looked at which genes were linked to the imaging-defined high-risk pattern, one gene called *NBPF4* stood out because it was supported by all 4 kinds of evidence: imaging features, gene expression, protein data, and genetic association studies. Follow-up experiments in cells and animals showed that boosting *NBPF4* activity made tumor cells grow faster, move more, form new lymphatic vessels, and spread to lymph nodes. Mechanistically, *NBPF4* worked by activating the mitogen-activated protein kinase (MAPK) signaling pathway and triggering a process known as epithelial mesenchymal transition (EMT). Interestingly, tumors with high *NBPF4* were sensitive to drugs that block one part of the MAPK pathway (JNK/p38) but resistant to another part (ERK), suggesting that the pathway had been rewired. Using this insight, computer-based drug screening and further testing identified MK-886 as a promising compound that could suppress *NBPF4*-promoted MAPK activation and tumor growth. Together, this work traces a complete path from a noninvasive imaging finding to a specific gene (*NBPF4*) and a potential treatment (MK-886). It establishes the *NBPF4*–MAPK–EMT axis as a key player in breast cancer metastasis and provides a general framework for turning imaging-based risk predictions into biological understanding and possible therapies.

## Introduction

Breast cancer (BRCA) axillary lymph node metastasis (LNM) is a well-established prognostic factor [1–4]. However, identifying high-risk patients preoperatively and developing effective therapies to counteract metastatic spread remain significant clinical challenges [4,5]. While deep learning models show increasing promise in predicting LNM from routine imaging [6–12], they often function as “black boxes”, failing to illuminate the underlying biological mechanisms, such as the cellular origins and molecular drivers, of the metastatic phenotypes they detect. In parallel, genomic and transcriptomic studies have identified many molecular alterations associated with metastasis [13–19], yet a major translational gap persists in refining these findings into biologically coherent, druggable targets and linking them back to clinically accessible imaging features.

To bridge this gap, integrative approaches are needed to systematically link noninvasive radiographic patterns with causal molecular mechanisms, thereby transforming imaging-based risk stratification into a discovery engine for novel biology and therapeutic targets. Here, we report a multi-stage radiogenomic pipeline designed to achieve this goal. This pipeline begins with a clinically motivated question: how to preoperatively identify patients at high risk of LNM. To address this, we first developed a deep learning Magnetic Resonance Imaging (MRI) model as a noninvasive screening tool to enrich for tumors with high metastatic potential. The imaging-defined high-risk phenotype then served as the entry point for multi-omics integration, which converged on *NBPF4* as the central molecular driver. Subsequent functional validation and drug screening completed the discovery-to-therapy cycle. We first developed a deep learning model for noninvasive LNM prediction and then integrated this imaging phenotype with multi-omics data to prioritize candidate driver genes. This approach identified *NBPF4* as a central hub gene supported by convergent evidence from transcriptomics, proteomics, and genetics. Mechanistically, *NBPF4* activated the mitogen-activated protein kinase (MAPK) signaling pathway and induced epithelial–mesenchymal transition (EMT), and we ultimately identified MK-886 as a candidate therapeutic compound capable of suppressing this *NBPF4*–MAPK–EMT axis that promotes​ LNM.

This study provides an integrative framework for generating mechanistic hypotheses and therapeutic leads regarding the molecular basis of imaging-defined metastatic risk in BRCA. Our work establishes a methodology for converting radiographic phenotypes into discoverable biological mechanisms and viable therapeutic strategies.

## Results

### Integrated radiogenomic pipeline predicts LNM and identifies a target gene

**Fig. 1.**
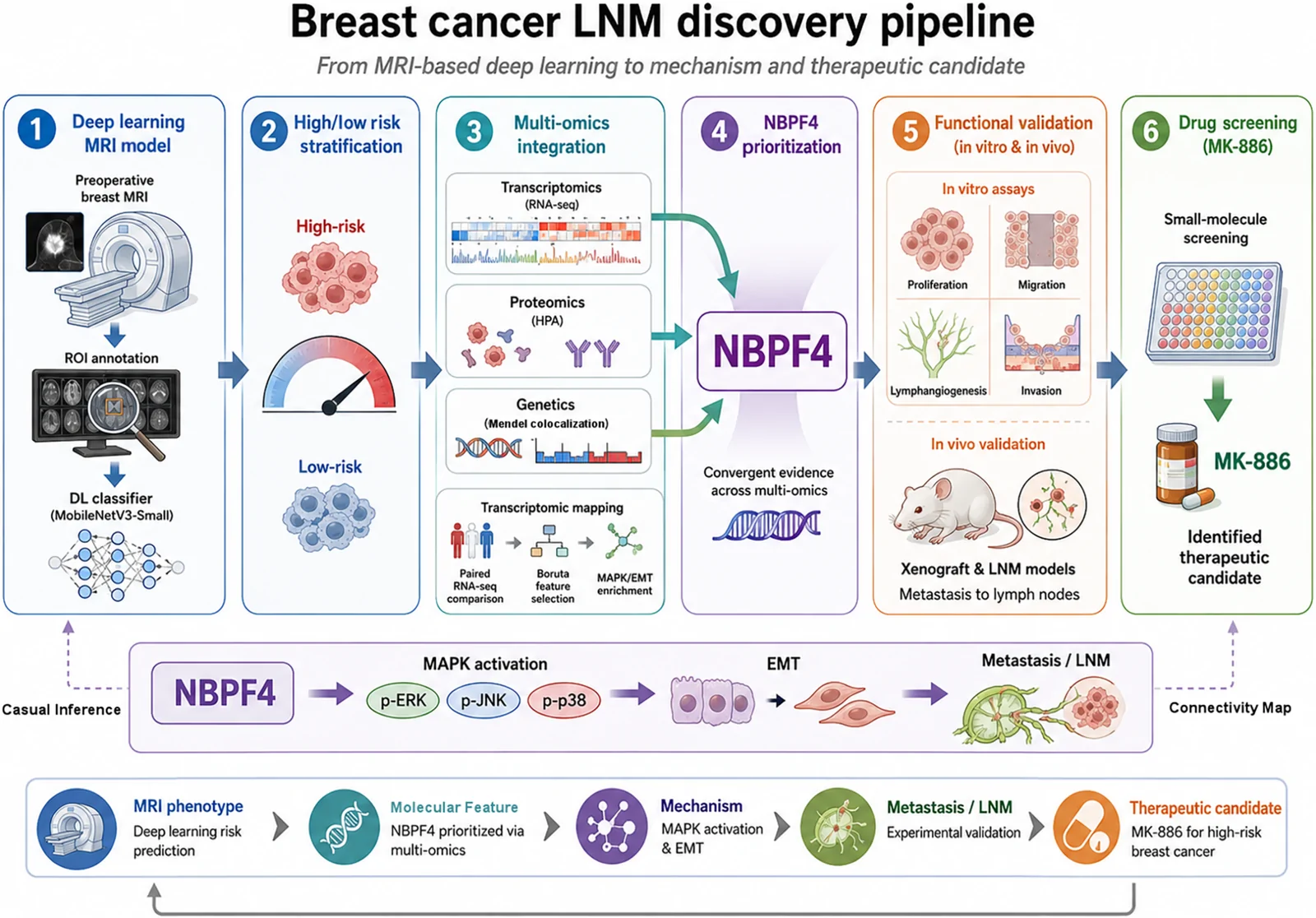
Integrated radiogenomic pipeline predicts LNM and identifies a target gene. Through a multicenter retrospective study, we developed a deep learning model for predicting LNM from preoperative BRCA MRI. Multi-omics analysis of high-risk patients identified the gene *NBPF4*. Causal inference using double LASSO and causal forest DML confirmed a significant effect of *NBPF4* on the imaging-defined risk phenotype, independent of genomic confounders.​ We further showed that *NBPF4* promotes metastasis by activating the MAPK pathway and EMT. Computational drug screening nominated MK-886, which inhibited the *NBPF4*–MAPK–EMT molecular mechanism. Our work establishes a closed-loop pipeline from imaging phenotype to therapeutic candidate.

This study established and validated a deep learning model based on the MobileNet V3 Small (MV3S) architecture for predicting the risk of LNM from preoperative contrast-enhanced breast MRI data in a multicenter retrospective study (Fig. 1). Multi-omics analysis of the high-risk group identified by the model prioritized candidate genes associated with MAPK signaling and EMT, leading to a focus on *NBPF4* for functional validation. Experiments demonstrated that overexpression of *NBPF4* promotes tumor cell proliferation, migration, and lymphangiogenesis, mediated through activation of the MAPK pathway and induction of EMT. Further computational drug screening identified MK-886, which was shown to inhibit *NBPF4*-associated MAPK pathway activity and tumor growth, confirming its therapeutic potential. Causal inference using double least absolute shrinkage and selection operator (LASSO) and causal forest double machine learning (DML) confirmed a significant effect of *NBPF4* expression on the imaging-defined high-risk phenotype, independent of genomic confounders, establishing *NBPF4* as an independent risk factor for increased imaging risk. This research constructs a complete framework from imaging phenotype to molecular mechanisms and therapeutic strategies.

### Deep learning predicts LNM and links imaging to pro-metastatic biology

**Fig. 2.**
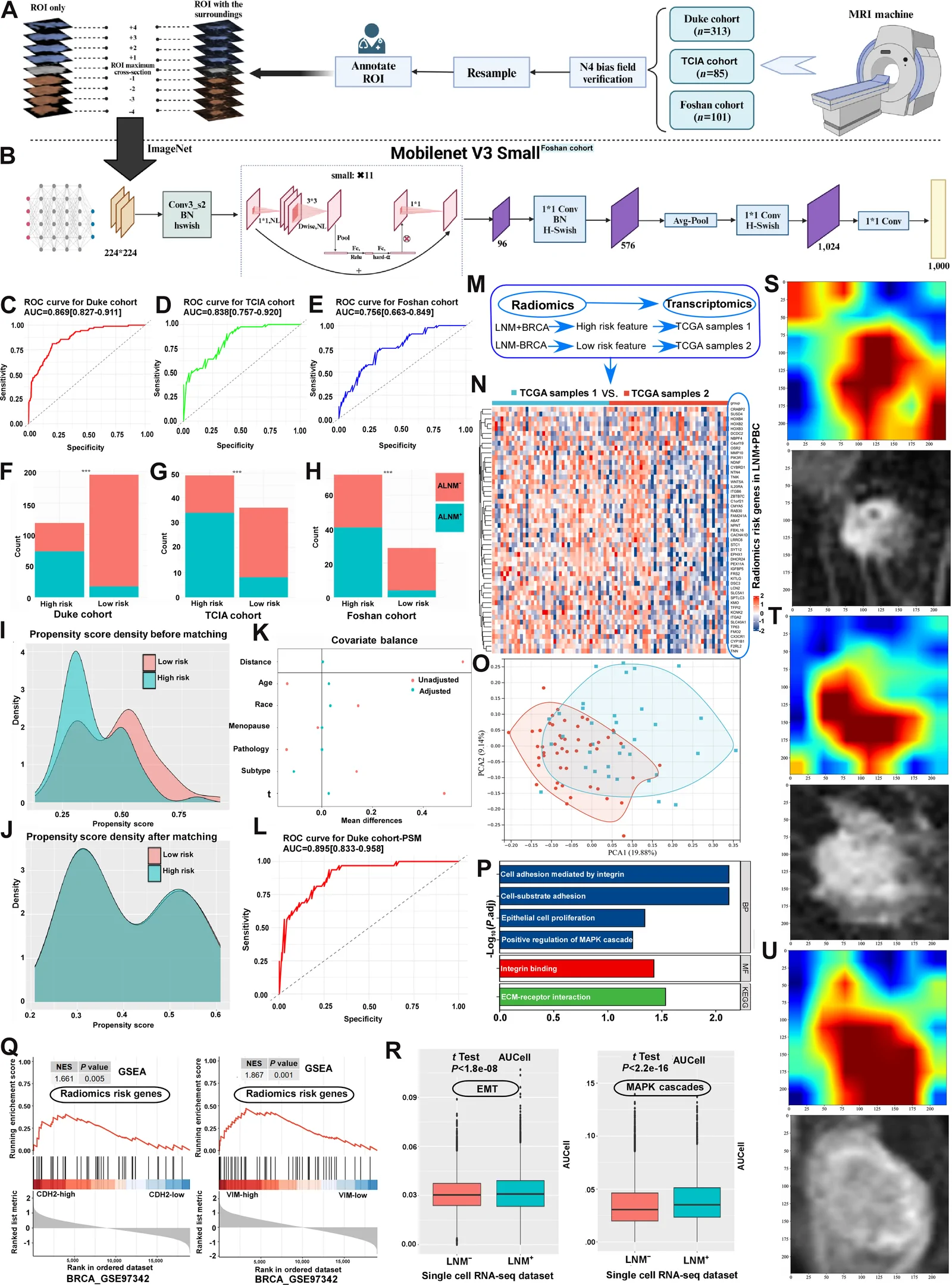
Deep learning predicts LNM and links imaging to pro-metastatic biology. (A) Schematic of the MRI preprocessing pipeline, encompassing region-of-interest (ROI) annotation, resampling, and bias field correction. (B) Architecture of the MV3S convolutional neural network used for feature extraction and risk prediction. (C to E) Receiver operating characteristic (ROC) curves demonstrating the performance of the MV3S model in stratifying patients into high- and low-risk groups across 3 independent cohorts: Duke cohort [area under the curve (AUC) = 0.869], The Cancer Imaging Archive cohort (TCIA; AUC = 0.838), and Foshan cohort (AUC = 0.756). (F to H) Stacked bar charts comparing the distribution of axillary lymph node (ALN) status between the model-predicted risk groups in each cohort (all *P* < 0.001, χ^2^ test). (I to L) Propensity score matching (PSM) analysis in the Duke cohort. (I and J) Density plots of propensity scores before (I) and after (J) matching. (K) Covariate balance plot showing improved similarity between matched high- and low-risk groups. (L) ROC curve of the MV3S model applied to the matched Duke cohort (AUC = 0.895). (M to P) Radiogenomic analysis linking imaging phenotypes to transcriptomic programs. (M) Workflow connecting high-risk imaging features from the Duke, Foshan, and TCIA cohorts to RNA-sequencing data from matched TCGA samples. (N) Heatmap of differentially high expressed genes linked to radiographic LNM risk (log_2_ fold change > 0, average expression > 1.5) between imaging-defined high- and low-risk TCGA samples. (O) Principal components analysis of the samples based on the expression profile of the differentially expressed genes, revealing a clear separation between the imaging-defined high- and low-risk groups. (P) Functional enrichment analysis (GO and KEGG) of the differentially expressed genes, highlighting significant activation of the MAPK signaling pathway and pathways related to EMT. (Q) GSEA plot for a custom EMT-related gene set. Samples were stratified into high (top 30%) and low (bottom 30%) expression groups based on the EMT marker *CDH2* or *VIM*. The positive normalized enrichment score (NES) indicates significant enrichment of the radiomics risk genes in the high-expression group, confirming that EMT pathway activation is associated with the high-risk radiographic phenotype. (R) Box plots visualizing EMT and MAPK cascade activity scores across distinct cell clusters in single-cell RNA-seq data, comparing tumors with (LNM^+^) and without (LNM^−^). Elevated scores in specific clusters of LNM^+^ samples indicate cell-type-specific activation of these pro-metastatic programs. (S to U) Attention overlay maps showing that the model-predicted LNM risk localizes to the tumor–stroma interface and heterogeneous tumor cores. The spatial pattern is consistent with the top SHAP-attributed features (margin irregularity, intratumoral heterogeneity, peritumoral enhancement). Experiments were repeated at least 3 times.

To develop a noninvasive tool for BRCA risk assessment, we constructed a deep learning pipeline based on preoperative MRI images. Following standardized preprocessing (Fig. 2A), we implemented an MV3S convolutional neural network for feature extraction and prediction (Fig. 2B). The model achieved robust performance in stratifying patients into high- and low-risk groups across 3 independent cohorts [Duke, area under the curve (AUC) = 0.869; The Cancer Imaging Archive (TCIA), AUC = 0.838; Foshan, AUC = 0.756; Table S1 and Fig. 2C to E]. The predicted risk groups were strongly associated with LNM^+^ status, a key clinical prognostic factor (Fig. 2F to H). To ensure the robustness of this association, we performed propensity score matching (PSM) on the Duke cohort, which balanced potential confounders and yielded an even higher predictive accuracy (AUC = 0.895; Fig. 2I to L).

To identify the biological basis of the imaging-derived risk phenotype, we then sought to interrogate the transcriptomic correlates of radiomic. Integration of imaging and transcriptomic data (Fig. 2M) yielded a set of 51 highly expressed genes. To ensure the robustness of this signature, we performed a rigorous feature selection using the Boruta algorithm, which confirmed 49 of these genes as stable, important predictors (Fig. S1). Their differential expression distinguished the risk groups, as confirmed by principal components analysis (Fig. 2N and O). Functional enrichment revealed that these genes were significantly enriched for pro-tumorigenic pathways, notably MAPK signaling and EMT regulation (Fig. 2P). To independently validate the link to EMT, we performed gene set enrichment analysis (GSEA) using an EMT gene set on samples stratified by the expression of canonical EMT markers (*CDH2* or *VIM*). The radiomics risk gene signature was significantly enriched in samples with high EMT marker expression (Fig. 2Q), corroborating the association. Finally, at single-cell resolution, we observed that EMT and MAPK pathway activity scores were significantly elevated in cancer clusters within LNM^+^ compared to LNM-BRCA (Fig. 2R), indicating that these pro-metastatic programs are activated in a malignant epithelial cell manner within the lymph node metastatic tumor ecosystem.

To quantitatively identify which imaging features drive the high-risk prediction, we performed SHapley Additive exPlanations (SHAP) attribution analysis on the radiomic feature set. The top-contributing features were measures of margin irregularity, intratumoral heterogeneity, and peritumoral enhancement, all of which are biologically plausible correlates of EMT and MAPK-driven aggressive behavior. Consistent with this, attention heatmaps revealed that our deep learning model primarily focuses on the tumor–stroma interface and heterogeneous tumor cores (Fig. 2S to U). These radiological “hotspots” correspond to the regions where the above high-contribution features are most salient.

This multi-modal analysis establishes a deep learning model for noninvasive LNM prediction and links the radiographic phenotype to MAPK/EMT signaling programs. In summary, the mapping from the MRI-derived risk phenotype to the transcriptomic gene signature and onward to causal inference proceeded as follows: (a) deep learning (DL)-based risk stratification of patients into high- and low-risk groups; (b) differential expression analysis between these groups using paired RNA-sequencing (RNA-seq) data; (c) Boruta-based feature selection to retain stable genes; (d) functional enrichment analysis linking the signature to MAPK/EMT pathways; and (e) causal inference using double LASSO to control for genomic confounders and Causal Forest DML to estimate the average treatment effect of *NBPF4* on Radrisk [average treatment effect (ATE) = 0.037, 95% CI: 0.005 to 0.070], establishing a statistically significant causal link from *NBPF4* expression to the imaging-defined high-risk phenotype.

### Multi-omic convergence validates *NBPF4* as an LNM-associated gene

**Fig. 3.**
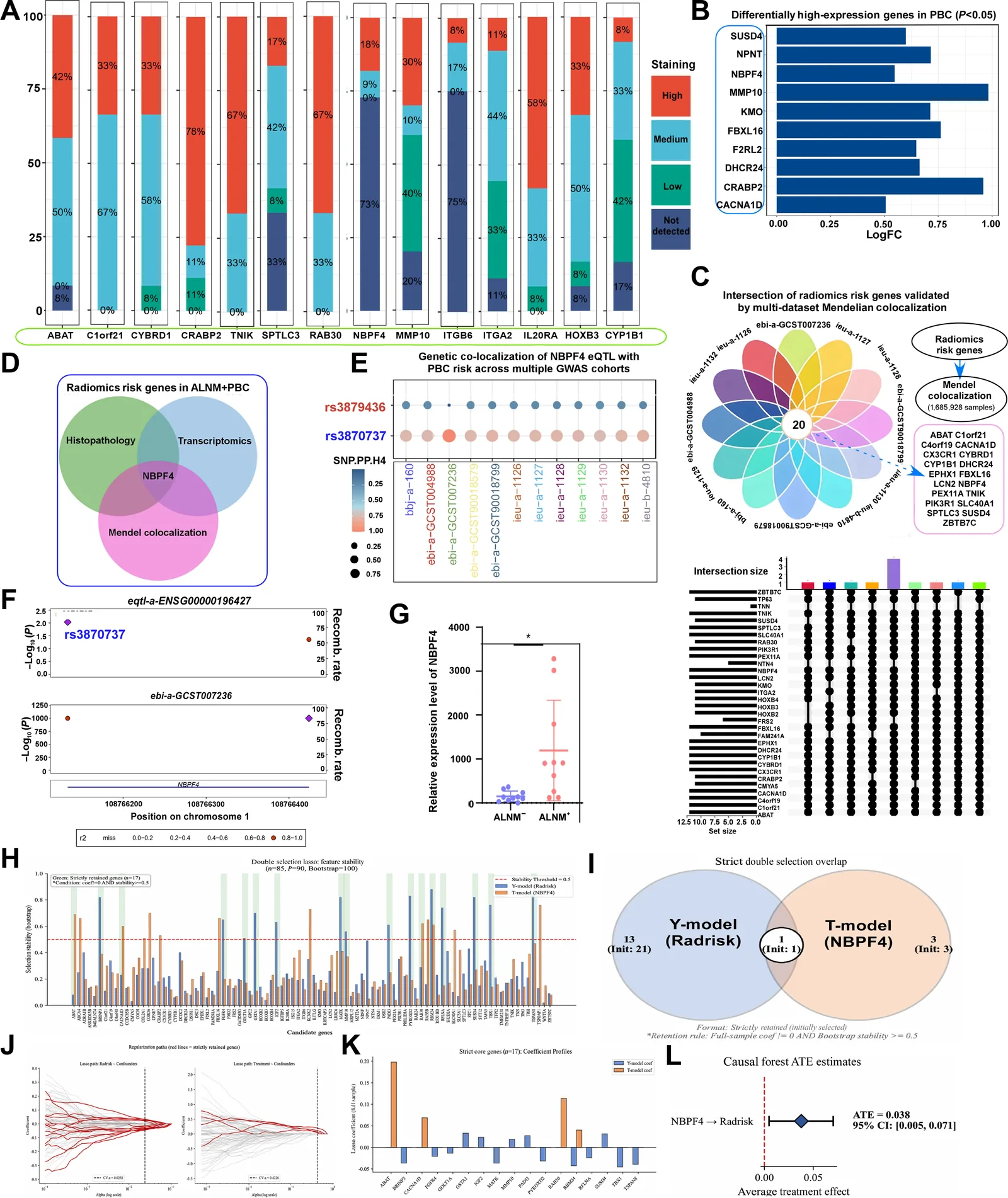
Multi-omic convergence validates *NBPF4* as an LNM-associated gene. (A) Stacked bar plots summarizing immunohistochemical staining patterns in breast tumors (Human Protein Atlas) for radiomics risk genes linked to LNM. Staining intensity is color-coded: red (high), blue (medium), green (low), and dark blue (not detected). Each bar represents the proportion of tumors in each staining category for a given gene, revealing heterogeneous expression profiles. (B) Horizontal bar graph of the top differentially up-regulated genes (logFC > 0.5, FDR < 0.05) in BRCA versus normal tissue from TCGA, ranked by log_2_ fold change in descending order. *NBPF4* is highlighted as a top 10 candidate. (C) Intersection analysis (Venn diagram and UpSet plot) of radiomics risk genes supported by SNP colocalization evidence across 12 independent BRCA GWAS datasets. (D) Venn diagram identifying the set of radiomics risk genes (*n* = 51) linked to LNM that are also supported by transcriptomic up-regulation in TCGA-BRCA (*n* = 10), high protein expression by IHC (*n* = 14), and genetic colocalization evidence (*n* = 19). *NBPF4* is the sole gene common to all 4 layers. (E and F) Cross-dataset validation of genetic colocalization at the *NBPF4* risk locus. (E) Regional association plot (gassocplot2) visualizing the colocalization of expression quantitative trait loci (eQTLs) and BRCA risk across 12 GWAS datasets, identifying a shared putative causal variant (rs3870737, PP.H4.abf > 0.8), supporting a shared genetic basis. (F) Regional association plot of eQTL-GWAS colocalization at the *NBPF4* locus in the representative cohort ebi-a-GCST007236. The lead variant rs3870737 (purple diamond) shows the strongest combined statistical evidence. (G) Validation of *NBPF4* overexpression in LNM^+^ BRCA. Box plot confirms elevated *NBPF4* mRNA levels in bulk tumor tissues from patients with LNM (LNM^+^) compared to those without (LNM-). Data are mean ± SEM; *P < 0.05, 2-sided *t* test. (H) Stability of differential genes in bootstrap of LASSO trajectories. (I) Feature selection process of the double LASSO algorithm. (J) Union of 2 models for screening genes. (K) Coefficient profiling of the selected genes as confounding factors. (L) Estimated ATE of causal effect of *NBPF4* on Radrisk. Experiments were repeated at least 3 times.

To substantiate the biological relevance of the radiomics risk gene set, we validated it across orthogonal molecular layers. Immunohistochemistry identified a subset with medium-to-high protein expression in clinical samples (Fig. 3A), while a complementary set of genes showed low or absent protein levels (Fig. S2), reflecting the heterogeneity of protein abundance within the radiomics risk signature. Transcriptomic analysis independently confirmed a subset that was significantly up-regulated at the mRNA level in BRCA [logFC > 0.5, false discovery rate‌ (FDR) < 0.05] (Fig. 3B).

To establish a genetic link, we performed Bayesian colocalization analysis across 12 BRCA genome-wide association study (GWAS) datasets. This identified a set of radiomics risk genes, harboring genetic variants that jointly influence gene expression and disease risk (Fig. 3C). Intersection analysis formally nominated *NBPF4* as the sole gene common to the radiomic, transcriptomic, proteomic, and genetic evidence layers (Fig. 3D). We further refined the genetic evidence, finding that the variant rs3870737 showed strong support (PP.H4 > 0.8) for being a shared causal variant across all 12 datasets (Fig. 3E), as detailed in the regional colocalization plots (Figs. S3 and S4). Detailed visualization at the *NBPF4* locus in a representative cohort confirmed the spatial overlap of expression quantitative trait locus (eQTL) and GWAS association signals. The colocalization evidence threshold was defined as PP.H4.abf > 0.8, consistent with standard practice (Fig. 3F). Finally, in an independent clinical cohort, *NBPF4* mRNA expression was significantly elevated in tumors from patients with pathologically confirmed LNM compared to those without metastasis (Fig. 3G).

Collectively, this sequential multi-omic integration, through a progression from radiographic phenotype to protein expression, transcriptomic dysregulation, population-level genetic association, and direct clinical validation, systematically nominates and validates *NBPF4* as a robust, multi-layer biomarker candidate functionally linked to the risk of LNM in BRCA.

Core confounders were identified via strict double LASSO. From the initial 90 candidate genes, target-specific LASSO regularization paths were constructed for both Radrisk and *NBPF4*. Bootstrap stability analysis demonstrated that a strict 0.5 threshold effectively filtered unstable features (Fig. 3H). After applying the dual-filtering criteria, 17 core genes exhibited high robustness across the LASSO trajectories (Fig. 3I). These variables were retained as control variables for subsequent causal inference (Fig. 3J). Coefficient profiling revealed that genes such as *ABAT* and *RAB30* strongly influenced the *NBPF4* model, whereas *RAB24* was the only gene that both greatly impacted the 2 models (Fig. 3K). We observed a significant positive causal effect of *NBPF4* on Radrisk. After rigorously controlling for the 17 core confounding genes, the Causal Forest DML model demonstrated a significant causal impact of *NBPF4* on Radrisk. The estimated ATE was 0.037 (95% CI: 0.005 to 0.070) (Fig. 3L). Because the 95% CI did not include zero, this causal effect was deemed statistically significant. These findings indicate that, independent of high-dimensional genomic confounders, elevated *NBPF4* expression is an independent risk factor for increased Radisk.

### *NBPF4* promotes BRCA growth in vitro and in vivo

**Fig. 4.**
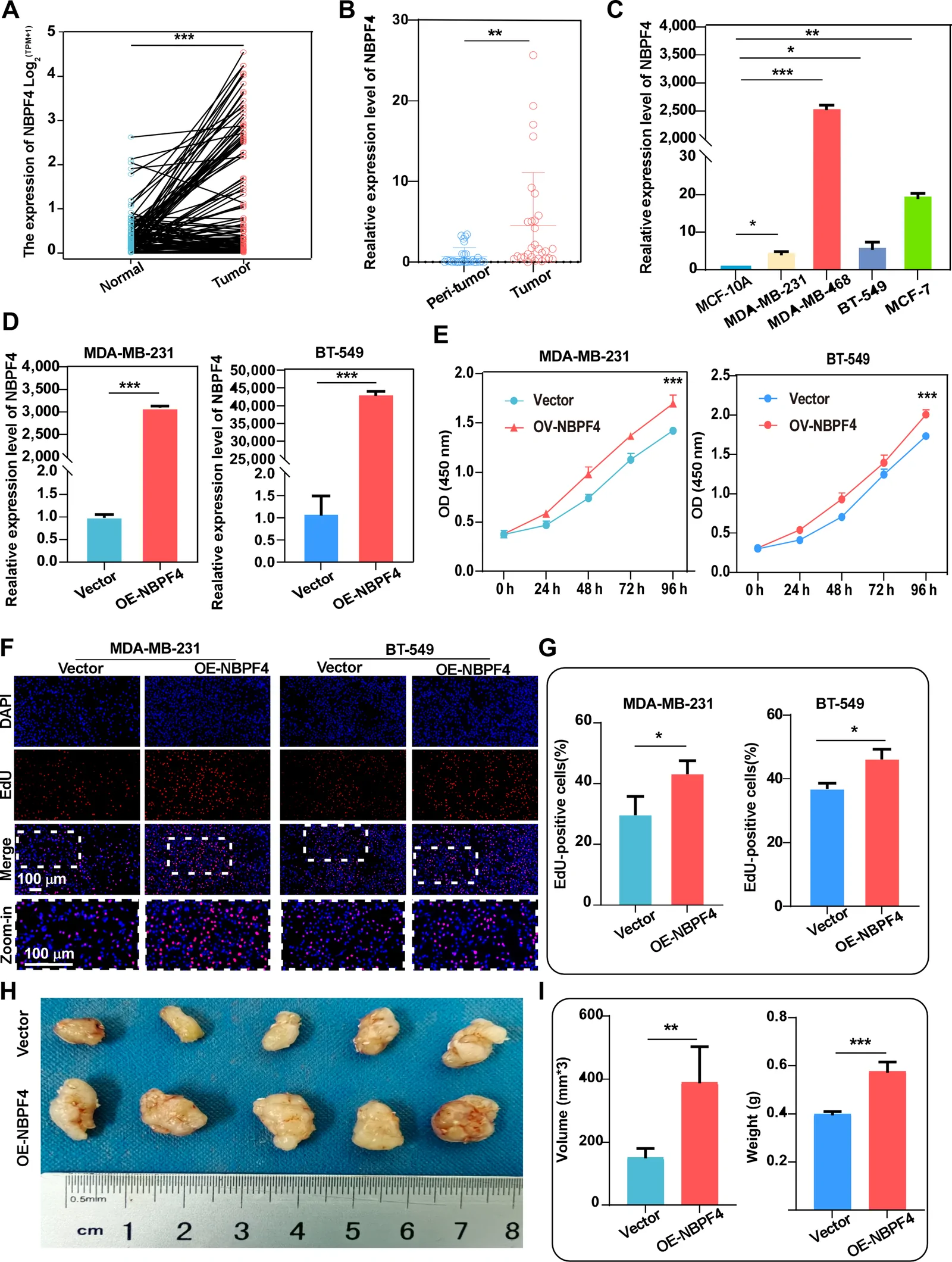
*NBPF4* promotes BRCA growth in vitro and in vivo. (A) Paired analysis of *NBPF4* mRNA expression in normal adjacent versus tumor tissues from the TCGA-BRCA cohort, confirming significant up-regulation in tumors. (B) Validation of *NBPF4* up-regulation by qRT-PCR in 30 independent matched patient samples (tumor versus peri-tumor tissue). (C) Relative *NBPF4* mRNA levels across a panel of breast cell lines, including the nontumorigenic MCF-10A line, showing elevated expression in cancer lines. (D) qRT-PCR confirmation of *NBPF4* overexpression (OE) efficiency in MDA-MB-231 and BT-549 cells transfected with an *NBPF4*-OE vector versus empty vector control. (E) Proliferation kinetics measured by CCK-8 assay in control and *NBPF4*-OE cells over 96 h. (F and G) Representative EdU immunofluorescence images [red, EdU; blue, 4′,6-diamidino-2-phenylindole (DAPI)] and quantification showing increased DNA synthesis in *NBPF4*-OE cells. (H and I) Tumor weights from subcutaneous xenografts derived from control or *NBPF4*-OE MDA-MB-231 cells harvested at endpoint. Tumor growth curves of the xenografts monitored over 28 days. Data are mean ± SEM. Statistical significance was determined by 2-tailed *t* test, paired *t* test, or 2-way analysis of variance (ANOVA) (*P < 0.05, **P < 0.01, ***P < 0.001). Experiments were repeated at least 3 times.

We next sought to characterize the functional role of *NBPF4*, a top candidate emerging from our multi-omics validation. Analysis of paired samples from the TCGA (The Cancer Genome Atlas)–BRCA cohort confirmed that *NBPF4* mRNA is significantly up-regulated in breast tumors compared to matched normal adjacent tissue (Fig. 4A). This finding was validated in an independent set of 30 patient-matched clinical samples (Fig. 4B). *NBPF4* expression was also elevated across a panel of BRCA cell lines compared to a nontumorigenic mammary epithelial cell line (Fig. 4C).

To assess its functional impact, we ectopically overexpressed *NBPF4* in 2 BRCA cell lines, MDA-MB-231 and BT-549 (Fig. 4D). *NBPF4* overexpression significantly enhanced cellular proliferation, as measured by both metabolic activity [Cell Counting Kit-8 (CCK-8) assay; Fig. 4E] and DNA synthesis [5-ethynyl-2′-deoxyuridine (EdU) incorporation; Fig. 4F and G]. The pro-tumorigenic function of *NBPF4* was further substantiated in vivo. Xenograft tumors derived from *NBPF4*-overexpressing cells grew significantly faster and formed larger end-point masses compared to tumors from control cells (Fig. 4H and I).

To contextualize the role of *NBPF4* beyond BRCA, we performed extensive pan-cancer analyses. *NBPF4* was frequently up-regulated at the transcriptomic and proteomic levels across multiple malignancies (Fig. S5A and B) and showed genetic associations with cancer risk in several GWAS (Fig. S5C). Its expression displayed notable tissue specificity, with highest levels in testis among normal tissues, and was detectable in immune cells and various cancer cell lines (Fig. S6). Importantly, *NBPF4* expression held prognostic value, capable of distinguishing tumor from normal tissue and associating with patient survival outcomes in a cancer-type-dependent manner, including in external BRCA cohorts (Fig. S7).

Collectively, these functional data move beyond association to establish a causal role for *NBPF4*. By demonstrating that its overexpression is sufficient to drive both in vitro proliferation and in vivo tumor growth, we define *NBPF4* as a core effector molecule that mechanistically links the radiogenomically defined risk phenotype to accelerated tumor progression, nominating it as a direct therapeutic target within this aggressive disease axis.

### *NBPF4* orchestrates an LNM pro-metastatic program

**Fig. 5.**
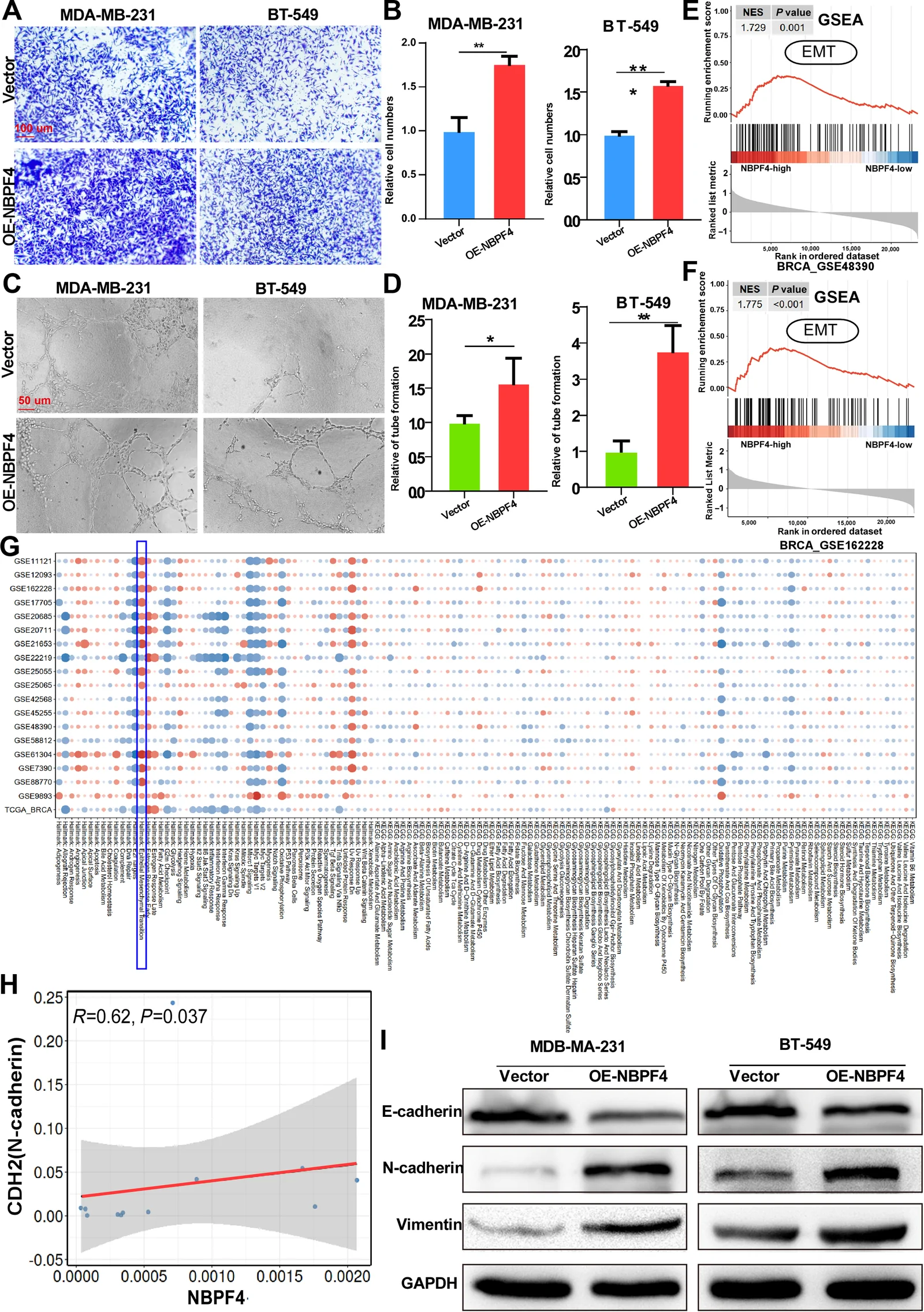
*NBPF4* orchestrates an LNM pro-metastatic program. (A and B) *NBPF4* enhances BRCA cell migration. Representative images (A) and quantification (B) of Transwell migration assays for control (Vector) and *NBPF4*-overexpressing (OE-*NBPF4*) MDA-MB-231 and BT-549 cells (scale bars, 100 μm). (C and D) *NBPF4* promotes LNM in vitro. Representative images and quantification of human lymphatic endothelial cell (HLEC) tube formation assays following incubation with conditioned medium from Vector or OE-*NBPF4* cells (scale bars, 100 μm). (E and F) *NBPF4* expression is associated with an EMT gene signature. Gene set enrichment analysis (GSEA) plots for a custom EMT-related gene set in 2 independent BRCA cohorts [(E), GSE48390; (F), GSE162228]. Samples were stratified by *NBPF4* expression (top versus bottom 30%). The positive NESs indicate significant enrichment of the EMT signature in *NBPF4*-high tumors. (G) Multi-pathway enrichment associated with high *NBPF4* expression. Bubble plot of GSEA results across multiple gene sets (Hallmark, KEGG). Bubble color indicates NES; size indicates statistical significance [−log_10_ (adjusted *P* value)]. (H) Positive correlation between *NBPF4* and the EMT marker *CDH2* across single-cell datasets. Scatterplot of mean *NBPF4* versus mean *CDH2* (N-cadherin) expression across multiple BRCA single-cell RNA-seq cohorts. Each point represents a dataset, colored by study. The Spearman correlation coefficient (*R*) and *P* value are indicated. (I) *NBPF4* overexpression modulates EMT-related protein expression. Western blot analysis of E-cadherin, N-cadherin, and Vimentin in control and *NBPF4*-overexpressing cells. GAPDH served as a loading control. Data are presented as mean ± SEM. (B, D, and H) Statistical significance was determined by 2-tailed *t* test (*P < 0.05, **P < 0.01, ***P < 0.001). Experiments were repeated at least 3 times.

We next investigated the functional mechanisms by which *NBPF4* might promote LNM. Ectopic overexpression of *NBPF4* significantly increased the migratory capacity of MDA-MB-231 and BT-549 cells in Transwell assays (Fig. 5A and B). Conditioned medium from *NBPF4*-overexpressing cells also potently enhanced the tube-forming capability of human lymphatic endothelial cells (HLECs) in vitro (Fig. 5C and D), suggesting a role in promoting lymphangiogenesis.

To understand the molecular basis of these phenotypes, we performed transcriptomic analyses. GSEA in 2 independent BRCA cohorts revealed that high *NBPF4* expression was significantly associated with the enrichment of EMT scores (Fig. 5E and F). Broad pathway analysis further confirmed the association of high *NBPF4* expression with multiple pro-tumorigenic processes, including EMT and inflammatory responses (Fig. 5G). This link to EMT was corroborated at the single-cell level, where a significant positive correlation was observed between the average expression of *NBPF4* and the canonical EMT marker *CDH2* (N-cadherin) across multiple independent single-cell RNA-seq datasets (Fig. 5H). At the protein level, NBPF4 overexpression led to a marked decrease in E-cadherin and concurrent increases in N-cadherin and Vimentin, confirming the induction of a full EMT program (Fig. 5I).

Collectively, this multi-faceted analysis demonstrates that *NBPF4* orchestrates a pro-metastatic program in BRCA cells, encompassing enhanced migration, promotion of a lymphangiogenic niche, and activation of the EMT transcriptional network, thereby providing a mechanistic foundation for its role in facilitating LNM.

### *NBPF4* links to MAPK pathway activation and drug response

**Fig. 6.**
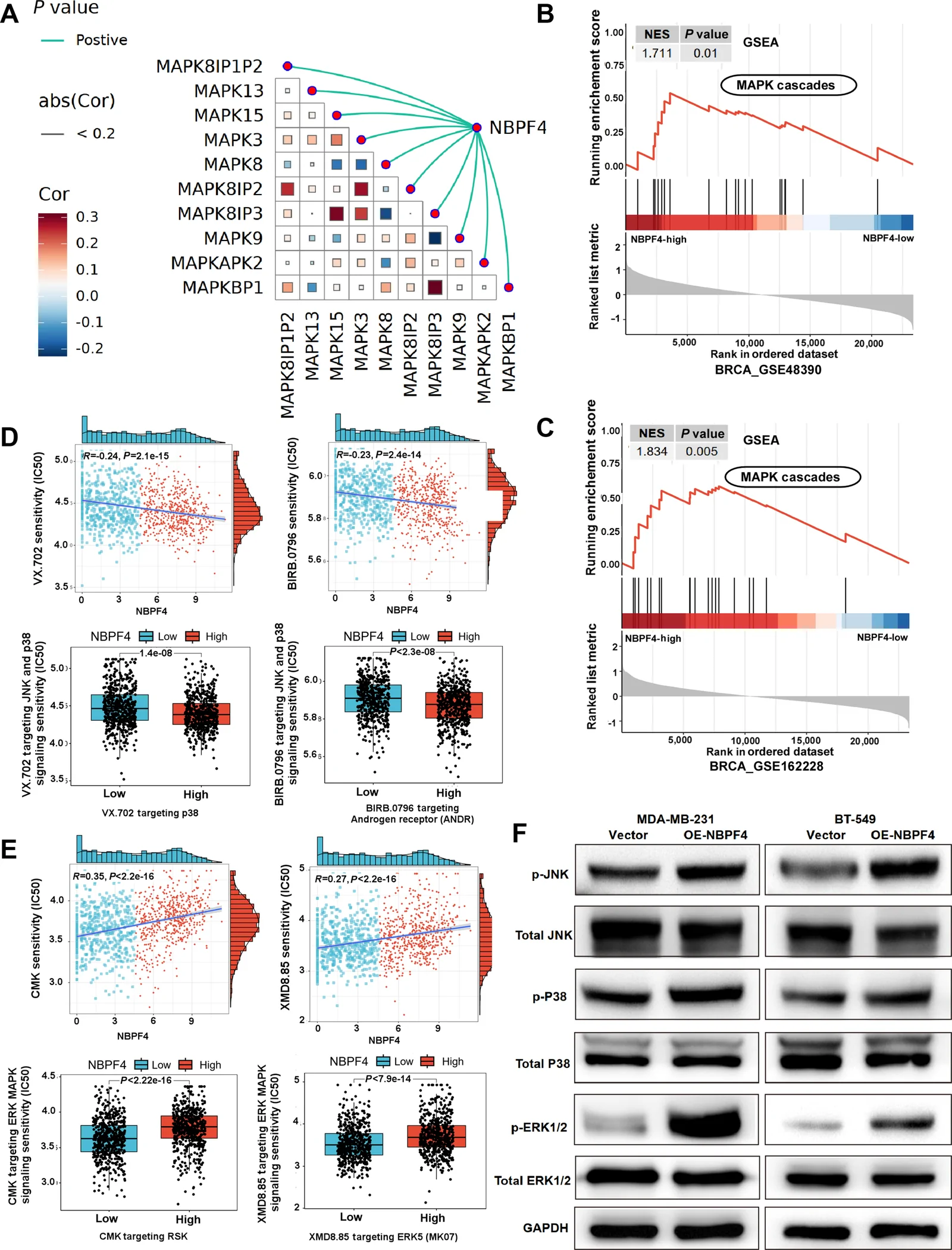
*NBPF4* links to MAPK pathway activation and drug response. (A) Correlation heatmap analyzing the relationship between *NBPF4* expression and MAPK-related genes in the TCGA-BRCA cohort. The heatmap displays a significant positive correlation (Spearman *ρ* = 0.48, *P* < 0.001), where increased color intensity indicates stronger association, demonstrating that elevated *NBPF4* expression is linked to enhanced MAPK signaling activity in breast tumors. (B and C) GSEA plots for a custom MAPK cascade pathway gene sets in 2 independent cohorts [(B) GSE48390; (C) GSE162228]. Samples were stratified by *NBPF4* expression (top versus bottom 30%). The positive NESs confirm significant enrichment of the MAPK signature in *NBPF4*-high tumors. (D and E) *NBPF4* expression predicts differential sensitivity to MAPK pathway inhibitors. (D) Comparison of the estimated half-maximal inhibitory concentrations (IC_50_) for MAPK pathway inhibitors targeting the JNK/p38 branches (VX.702, BIRB.0796) between *NBPF4*-high and *NBPF4*-low groups in the TCGA-BRCA cohort. (E) Comparison of the estimated IC_50_ for inhibitors targeting the ERK branch (CMK, XMD8.85) between the same groups. IC_50_ values were predicted from the GDSC1 database using the pRRophertic algorithm. Lower IC_50_ indicates higher drug sensitivity. The results show that *NBPF4*-high tumors exhibit enhanced sensitivity to JNK/p38 inhibitors but increased resistance to ERK-targeted inhibitors. *NBPF4* overexpression activates the MAPK pathway. (F) Western blot analysis of total and phosphorylated levels of key MAPK signaling components (ERK1/2, p38, JNK) in control and *NBPF4*-overexpressing cells, showing the strongest activation effect on ERK1/2 phosphorylation. GAPDH was used as a loading control. Data are presented as mean ± SEM. Statistical significance was determined by 2-tailed *t* test (*P < 0.05, **P < 0.01,***P < 0.001). Experiments were repeated at least 3 times.

To explore the mechanistic basis of *NBPF4’*s pro-tumorigenic role, we investigated its relationship with key oncogenic signaling. In the TCGA-BRCA cohort, *NBPF4* mRNA levels showed a significant positive correlation with a gene expression signature representing MAPK pathway activity (Fig. 6A). GSEA in 2 independent BRCA cohorts confirmed that tumors with high *NBPF4* expression were significantly enriched for MAPK pathway gene sets (Fig. 6B and C), indicating pathway activation in this context.

We next investigated the therapeutic implications of the association between *NBPF4* expression and MAPK pathway activity. Analysis of the GDSC1 drug sensitivity database using a ridge regression model (pRRophetic) revealed that BRCA patients with high *NBPF4* expression showed distinct response patterns to MAPK pathway inhibitors. Specifically, the *NBPF4*-high group demonstrated significantly increased sensitivity to inhibitors targeting the c-Jun N-terminal kinase (JNK)/p38 branches (VX.702, BIRB.0796) but exhibited marked resistance to extracellular signal-regulated kinase (ERK) pathway inhibitors (CMK, XMD8.85) compared to the *NBPF4*-low group (Fig. 6D and E). This differential drug sensitivity pattern was particularly intriguing given our protein-level validation results. Western blot analysis confirmed that *NBPF4* overexpression led to increased phosphorylation of key MAPK effectors, with particularly strong activation of ERK1/2, along with more moderate activation of p38 and JNK (Fig. 6F). The apparent paradox where strong ERK activation correlates with resistance to ERK-targeted therapy suggests that *NBPF4*-promoted tumors may undergo pathway rewiring, potentially developing alternative survival dependencies that reduce their reliance on the highly activated ERK branch while increasing vulnerability to JNK/p38 inhibition. These findings reveal that *NBPF4* not only activates the MAPK pathway but also orchestrates a therapeutic vulnerability shift, suggesting that *NBPF4* expression status could serve as a biomarker for guiding targeted therapy selection in BRCA, potentially favoring JNK/p38 inhibitors over ERK-targeted approaches in *NBPF4*-high tumors.

Collectively, these data establish a multi-level connection between *NBPF4* and MAPK signaling, spanning from transcriptional correlation and pathway enrichment to functional protein activation, and reveal a paradoxical yet therapeutically relevant pattern: High *NBPF4* expression predicts sensitivity to JNK/p38 inhibition but resistance to ERK inhibition. This branch-specific vulnerability positions *NBPF4* as a potential biomarker for guiding personalized MAPK-targeted therapy selection.

### *NBPF4* correlates with an aggressive oncogenic state and LNM in vivo

**Fig. 7.**
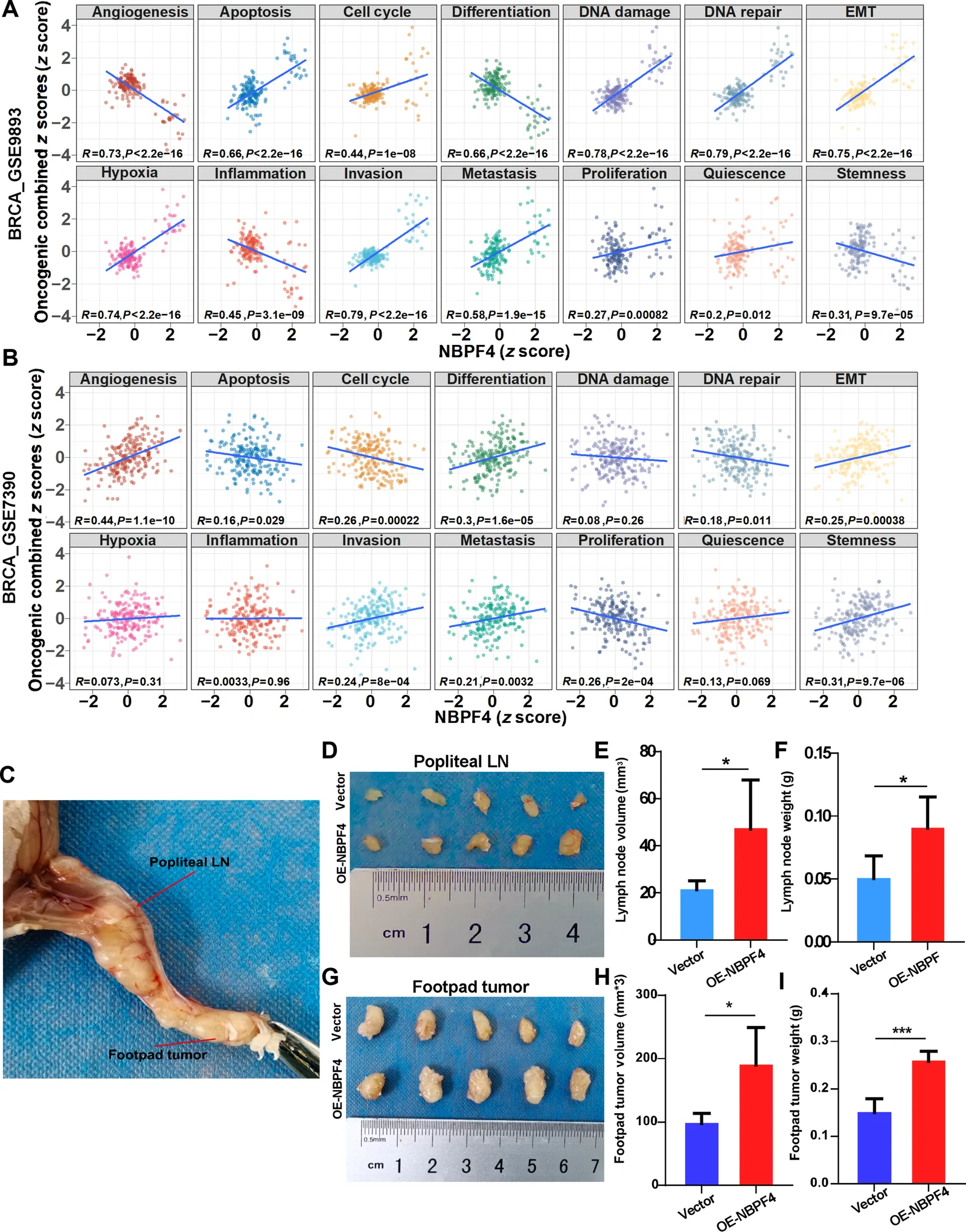
*NBPF4* correlates with an aggressive oncogenic state and LNM in vivo. (A and B) Correlation of *NBPF4* expression with oncogenic functional states in BRCA. Scatterplots depict the Pearson correlation between *NBPF4* gene expression (*x* axis, *z* score) and combined activity scores (*y* axis, *z* score) for 14 functional states (e.g., metastasis, EMT, and angiogenesis) in 2 independent BRCA cohorts: GSE9893 (A) and GSE7390 (B). Each point represents a tumor sample, color-coded by molecular or functional subtype. The Pearson correlation coefficient (*R*) and *P* value (2-tailed) are indicated for each state, revealing broad positive associations, most strongly with metastasis (GSE9893: *R* = 0.58, *P* = 1.9e−15; GSE7390: *R* = 0.21, *P* = 0.0032). (C to I) *NBPF4* promotes popliteal LNM in a mouse xenograft model. (C) Schematic and representative photograph of the experimental model, wherein MDA-MB-468 cells (vector control or *NBPF4*-overexpressing) were injected into the footpad of nude mice, with the popliteal lymph node as the primary draining site. (D) Representative images of excised popliteal lymph nodes from control and *NBPF4*-overexpressing (OE) groups. (E and F) Quantification of metastatic lymph node volume (E) and weight (F). (G) Representative images of excised primary footpad tumors. (H and I) Quantification of primary tumor volume (H) and weight (I). Data are presented as mean ± SEM. Statistical significance was determined by 2-tailed *t* test (*P < 0.05, ***P < 0.001). Experiments were repeated at least 3 times. For the mouse xenograft model, we assessed popliteal lymph node metastasis and therefore use the general term LNM rather than LNM, which is reserved for the clinical cohorts.

To systematically evaluate the functional context of *NBPF4*, we analyzed its association with 14 predefined oncogenic states (e.g., metastasis, EMT, and angiogenesis) across 2 independent BRCA transcriptomic cohorts. In both the GSE9893 and GSE7390 datasets, *NBPF4* expression showed significant positive correlations with multiple aggressive functional programs (Fig. 7A and B). The strongest and most consistent association was with the metastasis state (GSE9893: *R* = 0.58, *P* = 1.9e−15; GSE7390: *R* = 0.21, *P* = 0.0032), positioning *NBPF4* within a transcriptional program conducive to tumor dissemination.

We next sought causal, functional evidence for this correlative link using an in vivo popliteal LNM model. Injection of *NBPF4*-overexpressing (OE) MDA-MB-468 cells into the footpads of nude mice resulted in significantly larger and heavier metastatic lesions in the draining popliteal lymph nodes compared to vector-control cells (Fig. 7C to F). The primary footpad tumors in the *NBPF4*-OE group also exhibited increased growth, as measured by both volume and weight (Fig. 7G to I), confirming the pro-tumorigenic role of NBPF4 observed in prior in vitro assays.

Collectively, this figure integrates computational correlation with direct in vivo validation, demonstrating that *NBPF4* is transcriptionally linked to a metastatic functional state in human tumors and is sufficient to drive lymphatic metastasis and tumor growth in a preclinical model. This multi-modal evidence solidifies *NBPF4* as a functional mediator of BRCA progression and metastasis.

### Integrative discovery of MK-886 targeting the *NBPF4*-MAPK axis

**Fig. 8.**
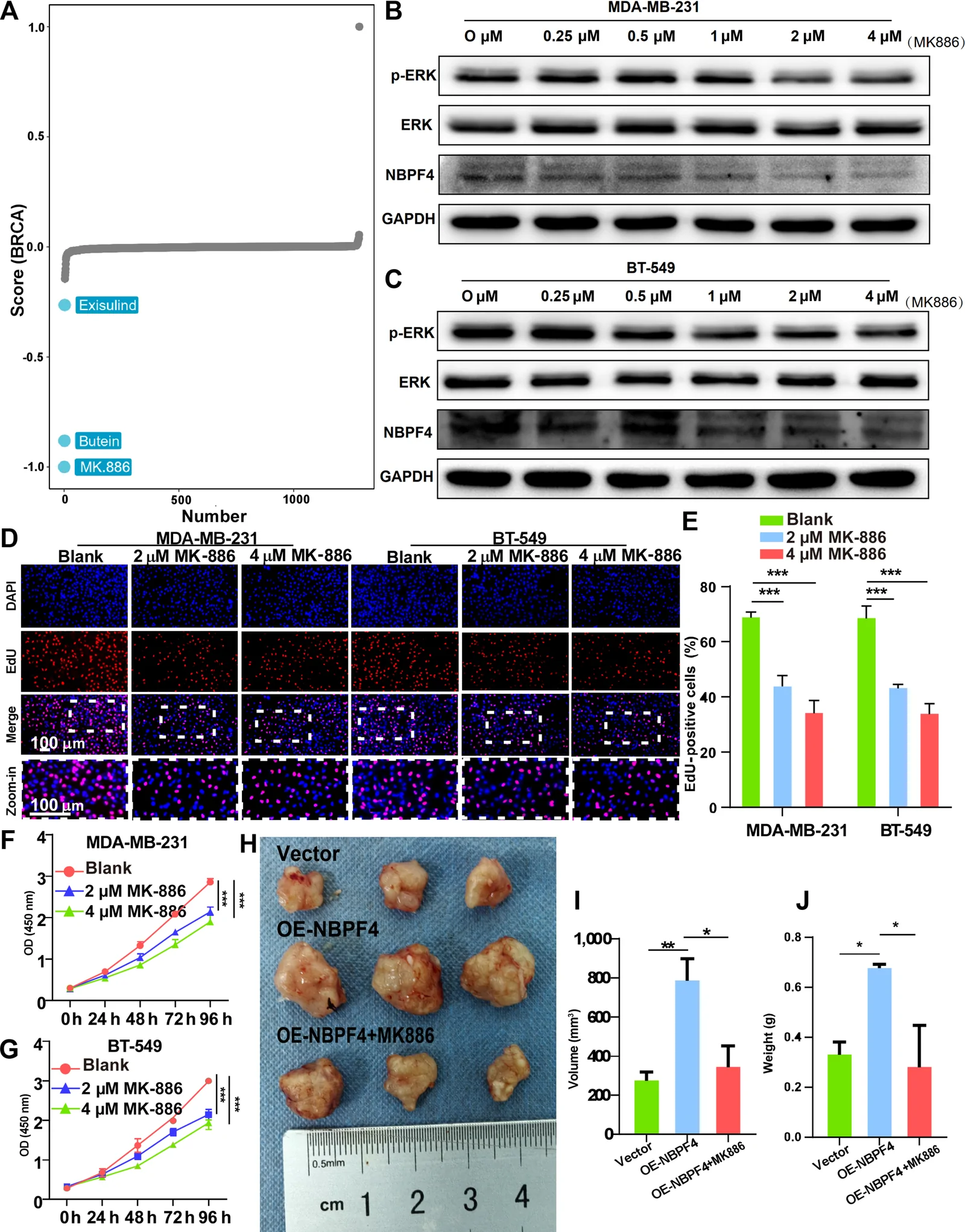
Integrative discovery of MK-886 targeting the *NBPF4*-MAPK axis. (A) Identification of MK-886 as a candidate inhibitor of *NBPF4*-mediated oncogenic signaling. Scatterplot showing the similarity scores for 1,288 compounds from the Connectivity Map (cMap) database, calculated using the XSum algorithm against a gene signature associated with high *NBPF4* expression. Lower scores (e.g., MK-886) indicate a higher potential to reverse the *NBPF4*-associated transcriptional profile. (B and C) MK-886 inhibits the *NBPF4*-mediated activation of the MAPK pathway. Western blot analysis of key MAPK pathway components (total and phosphorylated ERK1/2, p38, JNK) in control and *NBPF4*-overexpressing (OE) MDA-MB-231 (B) and BT-549 (C) cells treated with dimethyl sulfoxide (DMSO) (vehicle) or MK-886 (2 μM, 4 μM) for 24 h, followed by 48 h in drug-free medium. GAPDH serves as a loading control. MK-886 treatment reduces the elevated phosphorylation levels induced by *NBPF4* overexpression. (D and E) MK-886 suppresses *NBPF4*-enhanced cell proliferation in vitro. Representative EdU immunofluorescence images (D) and quantification (E) showing DNA synthesis in control and *NBPF4*-OE MDA-MB-231 cells treated with MK-886 (2 μM, 4 μM) for 24 h. Red, EdU; blue, DAPI. Scale bar, 100 μm. (F and G) Concentration-dependent anti-proliferative effect of MK-886. Cell viability assessed by CCK-8 assay in *NBPF4*-OE MDA-MB-231 cells treated with increasing concentrations of MK-886 (2 μM, 4 μM) for 12 h. (H and I) MK-886 attenuates *NBPF4*-promoted tumor growth in vivo. Tumor growth curves (H) and final tumor weights (I) of subcutaneous xenografts derived from control or *NBPF4*-OE MDA-MB-231 cells in nude mice. Mice were treated with vehicle or MK-886 (intraperitoneally) for 28 days. Data are presented as mean ± SEM. Statistical significance was determined by 2-tailed *t* test (I) or 2-way ANOVA (H) (*P < 0.05, **P < 0.01, ***P < 0.001). Experiments were repeated at least 3 times.

To identify potential therapeutics targeting NBPF4-mediated oncogenicity, we performed a pan-cancer Connectivity Map (cMap) analysis using the optimal XSum algorithm. Screening 1,288 compounds against an *NBPF4*-high gene signature across 24 cancer types identified MK-886 as a candidate with broad activity (Fig. S8). Subsequent BRCA-specific analysis confirmed MK-886 as a top candidate predicted to reverse the *NBPF4*-associated transcriptional profile (Fig. 8A).

We first tested whether MK-886 could counteract the molecular effects of *NBPF4*. Treatment with MK-886 markedly reduced the elevated phosphorylation levels of ERK1/2, p38, and JNK induced by *NBPF4* overexpression in both MDA-MB-231 and BT-549 cells (Fig. 8B and C), confirming its inhibitory effect on the *NBPF4*-activated MAPK pathway. Functionally, MK-886 treatment significantly decreased the enhanced DNA synthesis observed in *NBPF4*-overexpressing cells, as shown by EdU incorporation assays (Fig. 8D and E). Consistent with this, CCK-8 assays demonstrated a concentration-dependent suppression of cell proliferation by MK-886 (Fig. 8F and G). Finally, in a mouse xenograft model, administration of MK-886 potently attenuated the accelerated tumor growth driven by *NBPF4* overexpression (Fig. 8H and I).

Together, this integrated computational and experimental pipeline demonstrates that MK-886 effectively suppresses *NBPF4*-driven tumorigenesis by inhibiting the associated MAPK pathway activation, providing a preclinical rationale for targeting this axis in *NBPF4*-high expression.

## Discussion

The transformation of imaging patterns into mechanistic understanding remains a fundamental challenge in oncology, particularly in the context of metastasis where cellular plasticity and molecular adaptation drive progression [7,20–24]. Although current deep learning models can predict the risk of metastasis or treatment response based on imaging features, their primary function is to establish the correlation between these features and clinical outcomes. A key limitation is their inherent inability to elucidate the underlying causal mechanisms, such as the dynamics of cellular plasticity or the specific regulation of molecular pathways [25–27]. While deep learning has successfully decoded prognostic information from medical images, this advancement has inadvertently reinforced a diagnostic paradigm centered on correlation rather than causation [19,27–30]. The resultant “black box” problem is not merely an interpretability issue [31,32]. It represents a deeper methodological gap that separates descriptive phenotyping from the functional biology required for therapeutic intervention [33,34]. This study was designed to bridge this gap by establishing a closed-loop pipeline that explicitly connects an image-derived prognostic label to a functionally implicated molecular node and a candidate therapeutic strategy.

Recent radiogenomic research has predominantly focused on establishing parallel associations, correlating imaging features with molecular data [19,35–37]. While valuable, this approach often yields lists of candidate genes or pathways without a clear hierarchy of causality or a defined functional anchor [38]. Our methodology diverges from this convention by utilizing the imaging-derived risk phenotype as a functional screening tool. In this framework, the deep learning model functions as a selective filter, enriching for a tumor subpopulation whose defining biology is subsequently decoded through integrated multi-omics analysis. This causal prioritization led us to *NBPF4*, a gene whose role in BRCA metastasis was previously obscure. The convergence of transcriptional, proteomic, and genetic evidence around *NBPF4* validates this approach, demonstrating that imaging signatures, when properly leveraged, can pinpoint central regulatory nodes within a complex disease process. To further strengthen the causal link between the imaging phenotype and the molecular driver, we performed a double LASSO selection to control for genomic confounders and applied a Causal Forest DML model. The analysis revealed a significant positive association between *NBPF4* and the imaging risk score, providing statistical evidence consistent with a causal hypothesis at the population level. This result links the MRI-defined high-risk phenotype to *NBPF4*–MAPK–EMT activity, although direct spatial co-registration or interventional validation would be needed to establish a definitive causal chain.

The MAPK pathway and the EMT program are widely recognized as 2 central pillars driving cancer metastasis [39,40]. Activation of the MAPK cascade, particularly through the ERK and p38 branches, promotes tumor cell migration, invasion, and lymphangiogenesis across various malignancies, including esophageal squamous cell carcinoma, clear cell renal cell carcinoma, BRCA, and melanoma [41–46]. Concurrently, the EMT program facilitates metastatic dissemination in cancers such as nasopharyngeal carcinoma, cervical squamous cell carcinoma, oral squamous cell carcinoma, and pancreatic cancer [47–50]. These 2 pathways are frequently interconnected, as MAPK signaling can directly phosphorylate key EMT transcription factors, and their cooperative regulation is evident in cancers like gallbladder carcinoma and clear cell renal cell carcinoma [44,51]. Notably, prior research on *NBPF4* had overlooked its role in BRCA metastasis, a gap that highlights the novelty of our discovery.

In this context, our functional investigation reveals that *NBPF4* operates as a potent orchestrator of metastatic competence. It enhances proliferation, migration, and lymphangiogenesis in vitro and, critically, promotes LNM in vivo. Mechanistically, it converges on the MAPK pathway and the EMT program. However, a more nuanced finding emerged from the drug sensitivity analysis: *NBPF4*-high tumors displayed a paradoxical profile of ERK pathway activation coupled with ERK inhibitor resistance, alongside heightened vulnerability to JNK/p38 inhibition. This suggests that *NBPF4* does not merely amplify the MAPK cascade but actively rewires its signaling logic, potentially creating branch-specific oncogenic dependencies. This insight, which would be invisible to a purely correlative analysis, underscores the value of moving from phenotypic association to gene-centric functional dissection.

The translational potential of this integrated pipeline is exemplified by the identification of MK-886 as a candidate therapeutic agent. The ability of this compound to suppress *NBPF4*-induced phenotypes, including reduced MAPK phosphorylation and inhibition of in vivo tumor growth, provides a compelling preclinical proof-of-concept. More broadly, our work outlines a pragmatic translational strategy. This strategy involves initial population-wide risk stratification using a noninvasive imaging tool, followed by a focused molecular assay to identify the patient subset most likely to respond to a targeted agent such as MK-886. This tiered approach effectively balances clinical scalability with biological precision.

We acknowledge several limitations that chart a course for future work. A notable challenge is the high sequence homology between *NBPF4* and its family member *NBPF6*, which hindered specific knockdown via small interfering RNA (siRNA)/short hairpin RNA (shRNA) and complicated definitive rescue experiments. Therefore, establishing *NBPF4* as a strict genetic dependency will require more precise genetic tools, such as CRISPR-based approaches, in follow-up studies.​ Beyond this, the precise biochemical function of *NBPF4* and its direct molecular interplay with the MAPK pathway require further elucidation. The efficacy and safety profile of MK-886 must be rigorously established in advanced preclinical models. Furthermore, investigating whether this *NBPF4*-MAPK axis is a common feature in other aggressive carcinomas could reveal a broader therapeutic paradigm. A further limitation is that our retrospective biobank retains routine formalin-fixed and paraffin-embedded (FFPE) blocks without spatial orientation relative to preoperative MRI; consequently, direct voxel-level spatial co-registration between MRI-attention regions and molecular/immunohistochemical markers (e.g., *NBPF4*, p-ERK/p-p38, EMT, and lymphangiogenesis markers) could not be performed. The proposed MRI–*NBPF4*–MAPK–EMT axis should therefore be regarded as a hypothesis-generating model rather than a validated causal chain. The current evidence relies on population-level statistical inference (double LASSO + Causal Forest DML) and biological plausibility of the model-attended regions (tumor–stroma interface, heterogeneous cores), but lacks direct voxel-level spatial co-registration or in vivo imaging validation. Prospective studies with orientation-tracked specimens and/or spatial transcriptomics will be required to close this spatial evidence gap. Additionally, we did not perform small-animal MRI to directly assess whether *NBPF4* overexpression alters imaging phenotypes. Future studies incorporating in vivo imaging in xenograft models would provide more direct evidence for the causal link between *NBPF4* and imaging features. Until such spatial and imaging-based functional evidence is obtained, the causal interpretation of the MRI–*NBPF4*–MAPK–EMT axis should be considered provisional.

## Conclusion

In conclusion, this study demonstrates a principled framework for converting imaging-based prognostication into biological discovery and therapeutic hypothesis. By tethering a deep learning phenotype to a prioritized candidate gene and a candidate drug, we provide more than a new biomarker. We offer a proof-of-concept framework for a new type of translational research that starts with the patient’s clinical image, proceeds through multi-omics convergence and causal inference to identify a molecular driver, and culminates in a mechanistically grounded therapeutic strategy.

## Materials and Methods

### Study design

We implemented a multi-center retrospective study leveraging radiogenomics to assess the presence of LNM in BRCA in 3 steps. Firstly, we established and evaluated a DL model based on MRI to predict LNM in BRCA patients. Subsequently, multi-faceted genomic differential analysis and enrichment analysis were conducted on risk prediction groups based on this model.

### Datasets and participants

Radiological and clinical data for the Duke cohort and TCIA cohort were obtained from TCGA [52]. The data of the Foshan cohort originated from the First People’s Hospital of Foshan. Radiological and clinical data for the Duke cohort and the imaging and transcriptomic data derive from the same patient cohort and are paired by TCGA ID, with TCGA providing the clinical prognostic and transcriptomic data and TCIA providing the corresponding imaging data [52]. The data of the Foshan cohort originated from the First People’s Hospital of Foshan. Inclusion criteria were as follows: (a) patients with pathological diagnosis of BRCA and (b) patients who have undergone preoperative enhanced MRI scans. Image processing was the same as in our previous study [19]. MRI scans were acquired on 1.5- or 3.0-T scanners (GE/Siemens/Philips) across sites; typical sequences were axial pre- and post-contrast T1-weighted [repetition time (TR) ~ 400 to 700 ms, echo time (TE) ~ minimal, slice thickness 1.0 to 2.0 mm with ≤0.2 mm gap]. To reduce intersite variability, all images were resampled to isotropic 1 × 1 × 1 mm voxels, intensity *z*-normalized (by masked tumor/breast region), and rigidly aligned to a common orientation before feature extraction or model input. The methodological framework of this study was illustrated in Fig. 1. The final import of images was illustrated in Fig. 2A. This study was approved by our hospital and conducted under the guidance of the Declaration of Helsinki. Given the retrospective study design, informed consent was waived in Duke and TCIA cohort. This Foshan cohort involving human participants was reviewed and approved by the Ethics Committee of our hospital (approval number: FSYYY-EC-SOP-008-02.0-A09). The work has been reported in line with the STROCSS criteria [53]. In the statistical analysis, the 95% confidence intervals for the AUC were calculated using the DeLong method.

### Baseline characteristic

The baseline characteristic of the study was presented in Table S1. Our study analyzed datasets from 3 cohorts of BRCA patients: Duke cohort (*n* = 313), TCIA cohort (*n* = 85), and Foshan cohort (*n* = 101).

### Construction of deep learning model

We engineered a DL model, dubbed MV3S. The structural design and data intake process of the model were depicted in Fig. 2B. The average of the predicted probabilities of the multi-layered and multi-processed images for each training sample is the model’s final risk prediction. Using the Youden’s index from the training data, we stratified risk across the datasets, enabling targeted intervention strategies. To confirm the independence of risk grouping as a predictive factor, we applied PSM to mitigate confounding factors between the risk groups, aiming to minimize intersample variability with the help of the “MatchIt” package (method = “nearest”, ratio = 1). Expanded to include: region-of-interest (ROI) annotation method (manual delineation on axial post-contrast T1 images), data split strategy (Duke for training/internal validation; TCIA and Foshan as external test sets), and threshold selection [Youden index on training receiver operating characteristic (ROC)]

### Cell line and cell culture

Chinese Academy of Sciences Cell Bank in Shanghai provided several cells including MCF-10A, MDA-MB-231, MDA-MB-468, BT-549, and MCF-7. A Special medium for MCF-10A cells (Procell, China) was used to culture the MCF-10A cells. MDA-MB-231 and MDA-MB-468 cells were cultured in Dulbecco's modified Eagle's medium (DMEM; Gibco, China) supplemented with 10% fetal bovine serum (FBS; Cellmax, China), while BT-549 and MCF-7 cells were cultured in RPMI 1640 medium (Gibco, China) supplemented with 10% FBS (Cellmax, China). All cells were maintained at 37 °C in a 5% CO₂ atmosphere.

### Plasmid and cell transfection

We purchased pcDNA3.1-*NBPF4* from GenePharma (Suzhou, China) in order to achieve overexpression of the *NBPF4*. The cells were transfected with DNA at 60% to 80% confluency by using jetPRIME transfection reagent (Polyplus, USA). After 6 h of transfection, we will replace the culture medium and then cultivate in complete medium for 48 h.

### Extraction of RNA and quantitative real-time polymerase chain reaction

In order to extract total RNA from cells, we used TRIZOL reagent (Absin, China) based on the manufacturer’s instructions. The reverse transcription was performed with PrimeScript RT Reagent Kit (Takara, China). Quantitative real-time PCR (qRT-PCR) was performed using Vazyme ChamQ Universal SYBR qPCR Master Mix. Relative expression levels were calculated using the 2^−ΔΔCt^ method, with β-actin serving as the internal control.

### Western blot

We separated total protein extracts using 10% sodium dodecyl sulfate–polyacrylamide gel electrophoresis (SDS-PAGE). Electrophoretic gels were then converted into polyvinylidene difluoride (PVDF) membranes (0.45 μm, Millipore, USA), after that, add 5% nonfat dried milk and block for 2 h. Membranes were incubated with primary antibodies overnight at 4 °C, including mouse anti-glyceraldehyde-3-phosphate dehydrogenase (GAPDH) (1:5,000, Proteintech), rabbit anti-E-cadherin (1:2,500, Proteintech), rabbit anti-N-cadherin (1:1,000, ABconal), rabbit anti-Vimentin (1:1,000, ABconal), rabbit anti-ERK1/2 (1:2,000, Proteintech), rabbit anti-p38 (1:1,000, Proteintech), mouse anti-JNK (1:2,000, Proteintech), rabbit anti-p-ERK1/2 (1:1,000, Proteintech), rabbit anti-p-p38 (1:1,000, Proteintech), and rabbit anti-p-JNK1/2/3 (1:1,000, Proteintech). On the following day, we incubated the membranes at room temperature with a secondary antibody conjugated with horseradish peroxidase (HRP). In the next step, the membrane was washed 3 times for every 10 min. Images were acquired using the UVP/ChemStudio Imaging System (Analytik Jena AG, Germany).

### Cell Counting Kit-8

The CCK-8 assay utilized 96-well plates containing 2,000 cells in 100 μl of medium. Adding 10 μl of CCK-8 (APExBIO) to the medium, it was incubated at 37 °C for 2 h, an then at 450 nm, the wells’ absorbances were measured.

### 5-Ethynyl-2′-deoxyuridine

Proliferation of cells can be measured with EdU Cell Proliferation Kit with Alexa Fluor 555 (CX003, CellorLab). We plated 50% of cells into 24-well plates on the first day and then conducted EdU experiments on the following day. EdU proliferating cells and DNA-stained cells were examined under a confocal microscope. ImageJ can be used to count the total number and EdU-positive cells, and then calculate the percentage of EdU-positive cells.

### Transwell assay

Migration of cells was assessed in Transwell chambers (Corning, USA). On the top of the chamber, cells were plated in serum-free medium and on the bottom, where exist 600 μl medium with 20% serum. After 48 h, the sample was fixed for 1 h with 4% paraformaldehyde and then stained with 0.1% crystal violet for 30 min. The migrating cells are finally counted using ImageJ.

### HLEC tube formation assay

HLEC cells are derived from a commonly used cell line routinely preserved in the laboratory, which are cultured adherently in a dedicated complete endothelial cell culture medium at 37 °C with 5% CO₂. The experimental procedures for HLEC are as follows: The Matrigel matrix is pre-thawed overnight at 4 °C, spread into a 96-well plate at 50 μl per well, and then placed in a 37 °C incubator to polymerize for 30 min for later use. Next, the treated cell supernatant is collected and centrifuged to obtain the conditioned medium, which is temporarily stored at 4 °C. Afterward, HLECs are resuspended in the aforementioned conditioned medium, seeded onto the pre-coated Matrigel wells at a density of 1 × 10^4^ cells per well, and cultured at 37 °C with 5% CO₂ for 6 to 24 h. Finally, images are captured in multiple randomly selected fields under an inverted microscope at 40× magnification, and the number of junctions of intact lumens is counted using ImageJ software as the core quantitative indicator of the experiment.

### Statistical analysis

Continuous variables among groups were compared using the Welch’s *t* test, limma test, or Wilcoxon test. Categorical variables were analyzed with Fisher’s exact test for unpaired data and McNemar’s test for paired data. The AUC was utilized to evaluate the predictive performance for LNM. The DeLong test assessed differences between the 2 ROC curves. Bonferroni correction was applied for multiple comparisons to adjust the significance levels. Propensity scores were derived using logistic regression. All tests were 2-tailed, with statistical significance defined as a *P* value < 0.05. Statistical analyses were conducted using R (version 4.2.3) and Python (version 3.9.2).

### Single-cell RNA sequencing and analysis

Single-cell RNA-seq libraries were prepared using microwell-based barcoding followed by reverse transcription and PCR amplification according to the GEXSCOPE Single Cell RNA Library Kit protocol [54]. Libraries were sequenced on an Illumina NovaSeq 6000 platform (150-bp paired-end reads). Raw reads were processed with CeleScope to remove low-quality sequences, and Cutadapt (v1.17) was used for adapter and poly-A trimming [55]. Following unique molecular identifier‌ (UMI) and cell-barcode extraction, reads were aligned to the GRCh38 reference genome using STAR (v2.6.1a) [56]. Gene-level UMI counts were generated with featureCounts (v2.0.1) to produce the expression matrix [57]. Cell-type annotation was performed by evaluating the expression of canonical marker genes derived from differentially expressed genes (DEGs) in the SynEcoSys database. Cluster identities and marker expression were visualized using Seurat (v3.1.2) functions (DoHeatmap, DotPlot, VlnPlot).

### Pathway enrichment analysis

Functional enrichment of DEGs was performed using the “clusterProfiler” R package (v3.16.1) for Gene Ontology (GO) and Kyoto Encyclopedia of Genes and Genomes (KEGG) analyses [58]. Significantly enriched pathways were defined by an adjusted *P* value < 0.05.

### Gene set scoring with AUCell

The UCell R package (v1.1.0) was employed to compute gene set activity scores based on the Mann–Whitney *U* statistic, which ranks genes by expression level per cell. This method supports robust scoring across diverse and large-scale single-cell datasets. Cell differentiation trajectories were reconstructed using Monocle2 [59], with highly variable genes selected for pseudotemporal ordering. DDRTree was applied for feature selection and dimensionality reduction. Genes exhibiting bimodal “on–off” expression patterns were assessed for switch points along the trajectory.

### Mendelian colocalization analysis

Bayesian colocalization was used to evaluate whether genetic variants associated with 2 traits share a causal mechanism. Analysis was restricted to genomic regions harboring eQTLs for relevant genes, with evidence for colocalization defined as a posterior probability PP.H4.abf > 0.8. Results were visualized using the stack_assoc_plotfunction from the gassocplot2 package.

### Pan-cancer gene expression landscape

Normalized expression data [normalized transcripts per million (nTPM)] for protein-coding genes across 50 tissues were integrated from the Human Protein Atlas (HPA v23.0) and GTEx projects. Consensus transcript expression values were summarized as the maximum nTPM per gene across datasets. Results were visualized using lollipop plots.

### Drug sensitivity analysis​

Drug response data were obtained from the Genomics of Drug Sensitivity in Cancer (GDSC) database, which includes 367 compounds screened across 987 cell lines. The oncoPredict R package was used to model relationships between gene expression and half-maximal inhibitory concentration (IC_50_) values [60]. Negative correlations indicated that higher gene expression sensitized cells to a drug, whereas positive correlations indicated resistance.

### Differential drug sensitivity by *NBPF4* expression

The pRRophetic package implemented a ridge regression model to predict clinical chemotherapy response based on baseline gene expression and in vitro drug sensitivity data from the Cancer Genome Project (CGP) [61]. Model outputs included estimated IC_50_ values for genes of interest (e.g., *NBPF4*) in TCGA samples, enabling stratification of high versus low expression groups for drug sensitivity comparisons.

### Boruta feature selection

Boruta is an all-relevant feature selection wrapper algorithm that, by default, employs a Random Forest classifier. The method performs a top-down search for relevant features by comparing the importance of original attributes with the importance of “shadow” attributes—randomly shuffled copies of the original features—and iteratively eliminating irrelevant ones. In each iteration, the algorithm compares the importance of each real attribute with the maximum importance observed among the shuffled shadow attributes. Attributes with importance significantly lower than that of the shadow attributes are continuously removed. Conversely, attributes whose importance is significantly higher are labeled as “Confirmed”. The shadow attributes are recreated in every iteration. The algorithm terminates when only “Confirmed” attributes remain or when the maximum number of runs (maxRuns) is reached. In the latter case, some attributes may remain without a definitive decision and are labeled as “Tentative”. In summary, the Boruta algorithm enables the identification of the most diagnostically relevant features from a larger set of candidate genes.

### Correlation of average gene expression​

The average expression level of each of 2 specified genes was computed across multiple independent single-cell RNA-seq datasets. Pairwise association between the genes was then assessed using Spearman’s rank correlation coefficient [62,63].

### Gene set variation analysis

Gene set variation analysis (GSVA) is a nonparametric, unsupervised method that transforms a gene-level expression matrix into a pathway-level or gene set-level activity matrix. It calculates an enrichment score for each predefined gene set within each sample, without the need for prior differential expression analysis between sample groups. This transformation from gene-centric to pathway-centric data enhances the biological interpretability of the results.

The core analysis was performed using the gsva() function from the GSVA R package. The algorithm provides several method options for calculating enrichment scores. In this study, the *z*-score method was employed. This method, as described by Lee et al., calculates a combined *z* score to reflect the activity of a given biological pathway or functional state.

The kcdfparameter, which defines the kernel type for the nonparametric estimation of the cumulative distribution function, was set to “Gaussian”. This setting is appropriate for log_2_-transformed or otherwise continuous, normalized expression data.

### Application to cancer functional states

Gene sets representing 14 distinct functional states of tumor cells (e.g., invasion, metastasis, and proliferation) were compiled from the CancerSEA database. The GSVA zscore method was applied to the gene expression matrix using these 14 gene sets to calculate a corresponding enrichment score (combined *z* score) for each functional state in each sample. Subsequently, these raw enrichment scores were standardized across samples using the R scale () function to generate a final “gene set score” for each functional state. Finally, Pearson correlation was computed between the expression level of a gene of interest (e.g., *NBPF4*) and each of the standardized gene set scores to assess their associations [64–66].

## Ethical Approval

This study involving human participants was reviewed and approved by the Ethics Committee of our hospital (approval number: FSYYY-EC-SOP-008-02.0-A09, RYE2024122302, and LW2025016HA). Written informed consent was obtained from the Foshan cohort, while the Duke and TCIA cohorts were exempted from informed consent requirements due to their retrospective and de-identified nature.

## Data Availability

Publicly available datasets used in this study include TCGA-BRCA (https://portal.gdc.cancer.gov/), TCIA (https://www.cancerimagingarchive.net/), and GEO. Processed data, imaging risk scores, model weights, and code used for deep learning model training and multi-omics integration are available from the corresponding author (laijianguo@gdph.org.cn) upon reasonable request.
