# Supplementary material for: MRI-Based Deep Learning Guides Multi-Omics Discovery of NBPF4 as a Therapeutic Target for Breast Cancer Lymph Node Metastasis
Source: Research (Wash D C). 2026 Aug 24;9:1402. doi: 10.34133/research.1402 (PMC13500915; doi:10.34133/research.1402)
Supplement: Supplementary file 1 [file research.1402.f1.zip › Supplemental Material.docx]

**Supplemental Material**

**Supplementary Figures and Table**

**Supplementary Figure 1. Machine-learning-based feature selection refines the radiomics-associated gene signature.** A. Feature importance scores for candidate radiomics risk genes, ranked by the Boruta algorithm. Each box represents the distribution of importance scores (y-axis, 0-25) across iterations for a single gene (x-axis). Genes are categorized as confirmed (cyan, e.g., FMO2, TP63), tentative (yellow), or unimportant (red) based on comparison with the importance of permuted “shadow” features (horizontal dashed lines). B. Convergence of feature importance across 400 iterations of the Boruta algorithm. Each line tracks the importance score (y-axis) of a single gene over successive algorithm runs (x-axis). The stability of confirmed features (cyan lines) above the shadow thresholds (dashed lines) contrasts with the fluctuating or low scores of tentative (yellow) and rejected (red) features, demonstrating the robustness of the selection process.

**Supplementary Figure 2. Immunohistochemical profiling identifies a subset of radiomics risk genes with low or absent protein expression.** A-B. Bar plots showing the distribution of immunohistochemical staining intensities (negative, weak, moderate, strong) for proteins corresponding to the radiomics risk gene set. Each bar represents the percentage of samples within a given staining category for a specific gene. Panel A highlights examples such as TP63 and KITLG, which show a notable proportion of samples with low or negative staining, indicating heterogeneity or low-level expression for this subset of genes. Panel B displays genes for which the majority of samples exhibit moderate-to-strong staining. This comprehensive profiling demonstrates variable protein abundance across the identified radiomics risk gene set.

**Supplementary Figure 3. Bayesian colocalization analysis was performed across 6 independent GWAS datasets of breast cancer.** Regional association plots illustrating the genetic co-localization of the *NBPF4* expression quantitative trait locus (eQTL) with breast cancer risk signals across 6 independent GWAS GWAS datasets of breast cancer. For each dataset, the-log10 (P-value) of genetic association is plotted against the genomic position on chromosome 1 (GRCh37/hg19). The lead eQTL SNP rs3870737 is denoted by a purple diamond, and all other SNPs are colored by their linkage disequilibrium (r²) with this lead variant. The local recombination rate is indicated by the blue line. High posterior probability for co-localization (PP.H4 > 0.8) supports the presence of a shared causal variant influencing both *NBPF4*expression and breast cancer risk at this locus.

**Supplementary Figure 4. Bayesian colocalization analysis was performed across 5 independent GWAS datasets of breast cancer.** Regional association plots for *NBPF4* across 5 additional GWAS datasets of breast cancer, confirming the reproducibility of the co-localization signal observed in Supplementary Fig. 3. Each plot displays the-log10 (P-value) of genetic association against the genomic position on chromosome 1. The lead eQTL SNP rs3870737 (purple diamond) consistently shows the strongest association. The high posterior probability for co-localization (PP.H4 > 0.8) across all nine independent datasets provides robust genetic evidence that variants regulating *NBPF4* expression are also causally linked to breast cancer susceptibility.

**Supplementary Figure 5. Pan-cancer landscape of *NBPF4* expression and genetic association.** A. Box plot comparing *NBPF4*mRNA expression (log2[TPM+1]) between tumor (T) and adjacent normal (N) tissues across 33 TCGA cancer types. Each point represents a sample; center line, median; box limits, IQR; whiskers, 1.5×IQR. Significant upregulation in tumors is observed in several cancer types, including breast invasive carcinoma (BRCA). B. Heatmap summarizing immunohistochemical staining patterns for the *NBPF4* protein across 20 human cancers from the Human Protein Atlas. Each row represents a cancer type, colored by the percentage of samples with high (red), medium (orange), low (green), or not detected (blue) staining intensity. Heterogeneous protein expression is evident across malignancies. C. Forest plot summarizing the genetic co-localization evidence for *NBPF4*across multiple cancer genome-wide association studies (GWAS). The posterior probability (PP.H4) that shared genetic variants influence both *NBPF4* expression and cancer risk is shown for each cancer type, with horizontal lines representing 95% credible intervals. This provides orthogonal genetic support for the role of *NBPF4* in carcinogenesis beyond breast cancer.

**Supplementary Figure 6. Expression atlas of *NBPF4* across normal tissues, immune cells, and cancer cell lines.** A. Violin plot of *NBPF4* normalized transcripts per million (nTPM) expression across 54 normal human tissues (GTEx/HPA). Testis shows exceptionally high expression. B. Dot plot of *NBPF4* nTPM expression across 18 major immune cell types. Expression is generally low but detectable in several subsets, including total PBMCs and T-regulatory cells. C. Heatmap of *NBPF4* nTPM expression across a panel of 60 cancer cell lines from the Cancer Cell Line Encyclopedia (CCLE). Variable expression is observed, with notably high levels in certain melanoma, leukemia, and breast cancer lines.

**Supplementary Figure 7. Prognostic and predictive value of *NBPF4* expression in pan-cancer and external cohorts.** A. Receiver operating characteristic (ROC) curve evaluating the ability of *NBPF4* expression to distinguish tumor from normal tissue in the TCGA pan-cancer cohort (Area under the curve [AUC] = 0.89, 95% CI 0.85-0.92). B. Results of univariate Cox proportional hazards regression for overall survival (OS) across 20 TCGA cancer types. Each bar represents the hazard ratio (HR) for high vs. low *NBPF4* expression; error bars denote 95% confidence intervals. Cancer types with significant association (P < 0.05) are highlighted, revealing both protective (HR < 1, e.g., ACC) and risky (HR > 1, e.g., COAD) associations. C-D. Kaplan-Meier survival curves for relapse-free survival (RFS, c) and overall survival (OS, d) in the GSE9893 breast cancer cohort, stratified by median *NBPF4* expression. High *NBPF4*expression is associated with significantly poorer RFS (log-rank P < 0.001) but, in this cohort, with better OS (log-rank P < 0.001), highlighting context-dependent prognostic effects.

**Supplementary Figure 8. Pan-cancer Connectivity Map (CMap) screening identifies MK-886 as a candidate *NBPF4* inhibitor.** A. CMap analysis was performed using the XSum algorithm to prioritize compounds predicted to reverse the transcriptional signature associated with high *NBPF4*expression. A. Heatmap displaying the XSum scores (ranging from 0 to-1, with darker blue indicating stronger predicted profile reversal) for 1288 compounds across 24 human cancer types. Each row represents a cancer type (e.g., BRCA, COAD, LUAD), and each column represents a compound. The top candidate compounds, including MK-886, consistently show strong negative scores (blue) across multiple cancers. Bar graph summarizing the screening results, showing the number of cancer types in which each compound yielded a negative XSum score (i.e., predicted reversal of the *NBPF4* signature). MK-886 (arrow) reversed the signature in the broadest number of cancer types, prioritizing it for subsequent breast cancer-specific validation in Figure 8A.

**Table S1** **Baseline data of the Duke cohort, TCIA cohort and Foshan cohort**

| **Characteristic** | **Duke cohort No.(%)** | **FoShan cohort No.(%)** | **TCIA cohort No.(%)** |
| --- | --- | --- | --- |
| No. of patients | 313(100) | 101(100) | 85(100) |
| Age | 54.81±10.47 | 51.11±10.00 | 54.89±10.93 |
| T stage |  |  |  |
| T1 | 166(53.0) | 63(62.4) | 30(35.3) |
| T2 | 129(41.2) | 38(37.6) | 50(58.8) |
| T3/T4 | 18(5.8) | 0 | 5(5.9) |
| N stage |  |  |  |
| N0 | 223(71.3) | 56(55.4) | 42(49.4) |
| N1 | 72(23.0) | 39(38.6) | 43(50.6) |
| N2 | 11(3.5) | 5(5.0) | 0 |
| N3 | 7(2.2) | 1(1) | 0 |
