# Supplementary material for: MRI-Based Deep Learning Guides Multi-Omics Discovery of NBPF4 as a Therapeutic Target for Breast Cancer Lymph Node Metastasis
Source: Research (Wash D C). 2026 Aug 24;9:1402. doi: 10.34133/research.1402 (PMC13500915; doi:10.34133/research.1402)
Supplement: Supplementary file 1 [file research.1402.f1.zip › Table S1.docx]

**Table S1.** **Baseline data of the Duke cohort, TCIA cohort and Foshan cohort**

| Characteristic | Duke cohort No.(%) | FoShan cohort No.(%) | TCIA cohort No.(%) |
| --- | --- | --- | --- |
| No. of patients | 313(100) | 101(100) | 85(100) |
| Age | 54.81±10.47 | 51.11±10.00 | 54.89±10.93 |
| T stage |  |  |  |
| T1 | 166(53.0) | 63(62.4) | 30(35.3) |
| T2 | 129(41.2) | 38(37.6) | 50(58.8) |
| T3/T4 | 18(5.8) | 0 | 5(5.9) |
| N stage |  |  |  |
| N0 | 223(71.3) | 56(55.4) | 42(49.4) |
| N1 | 72(23.0) | 39(38.6) | 43(50.6) |
| N2 | 11(3.5) | 5(5.0) | 0 |
| N3 | 7(2.2) | 1(1) | 0 |
